# Immunologic aspect of ovarian cancer and p53 as tumor antigen

**DOI:** 10.1186/1479-5876-3-34

**Published:** 2005-09-15

**Authors:** HW Nijman, A Lambeck, SH van der Burg, AGJ van der Zee, T Daemen

**Affiliations:** 1 Dept. of Gynaecologic Oncology, Groningen University Medical Center; 2 Dept. of Medical Microbiology, Molecular Virology Section, Groningen University Medical Center; 3 Dept. of Immunohematology and Blood Transfusion, Leiden University Medical Center

## Abstract

Ovarian cancer represents the fifth leading cause of death from all cancers for women. During the last decades overall survival has improved due to the use of new chemotherapy schedules. Still, the majority of patients die of this disease. Research reveals that ovarian cancer patients exhibit significant immune responses against their tumor. In this review the knowledge obtained thus far on the interaction of ovarian cancer tumor cells and the immune system is discussed. Furthermore the role of p53 as tumor antigen and its potential role as target antigen in ovarian cancer is summarized. Based on the increased knowledge on the role of the immune system in ovarian cancer major improvements are to be expected of immunotherapy based treatment of this disease.

## Introduction

Ovarian cancer is the most common cause of death from gynecological malignancies. Its nonspecific clinical presentation and the absence of effective screening methods are responsible for the 70% of patients who present with an advanced stage of disease at the time of diagnosis. Primary treatment for advanced stage ovarian cancer is cytoreductive surgery followed by platinum/paclitaxel based chemotherapy. An aggressive surgical approach has been advocated with the intent to remove all macroscopic disease which should yield better survival than leaving residual disease [1-3]. Response rates to primary chemotherapy are 65–80%. When residual or recurrent disease manifests itself, resistance to chemotherapy will prohibit further curative therapy, resulting in an overall survival for patients with advanced stage ovarian disease of only 10–20%[4,5].

Research during the last decades has revealed that ovarian cancer patients exhibit significant immune responses against their tumor (reviewed in this paper). In designing alternative treatments to successfully eradicate ovarian cancer it is important to consider both the positive effects of immune responses to ovarian cancer and the confounding negative effects on the immune system caused by the tumor cells. As the main target for a potential vaccine is the (overexpressed / mutated) p53 protein we will focus on studies aimed at the induction of humoral and cellular responses against this antigen. However, before reviewing these studies we will briefly introduce some general aspects of the cellular immune system including antigen encounter, antigen processing and presentation and factors influencing the outcome of the immune response in ovarian cancer.

## General introduction on the cellular immune system

Antigen presenting cells, most likely dendritic cells, can capture tumor antigens that are secreted or shed by tumor cells or by taking up dying tumor cells. The tumor antigens are processed and presented as peptides by major histocompatability complex (MHC) I and II molecules on the cell surface, and recognized by the T-cell receptor on T-cells. This phenomenon is often referred to as the first signal of activation. After cleavage of proteins into peptides by the proteasome complex and loading of peptides into the class I molecules in the endoplasmatic reticulum, these MHC class I – peptide complexes, recognized by cytotoxic T lymphocytes, are transported to the cell surface. MHC class II molecules mainly present exogenous endocytosed proteins. Antigen (peptide) loading of MHC class II molecules occurs within the endocytic pathway (MHC class II compartments). MHC class II – peptide complexes expressed on the cell surface are recognized by the CD4+ T helper cells. Next to this first antigen specific signal there is a need for a second signal. This signal involves the ligation of CD28 or CTLA-4 on lymphocytes by co-stimulatory molecules CD80 (B7.1) or CD86 (B7.2) respectively on antigen presenting cells or target cells. Binding of the CD28 receptor results in proliferation and activation of T cells, in contrast to binding of CTLA-4 which results in T cell anergy. Another important co-activation signal is mediated by interaction of CD40 ligand on T cells and CD40 on the antigen presenting cell. Fully activated CD8+ T cells differentiate into cytotoxic T lymphocytes and can lyse tumor cells. Memory CD4+ and CD8+ T cells play a critical role in maintaining protective immunity. Apart from their role in expanding CD8+ T-cells, CD4+ T-cells are also involved in the activation of CD8+ independent tumoricidal mechanisms which may play a role in the eradication of tumor cells that have lost MHC class I expression [6]. The CD4+ T cells can be divided into at least two subsets of T helper cells (Th), designated Th1 and Th2. Whereas a Th1 type immune response generally stimulates the generation of cellular immunity, a Th2 type response stimulates humoral immunity next to growth and differentiation of mast cells and eosinophils. Th1 cells secrete cytokines like IFN-γ, Il-2 and TNF-α, Th2 type cells mainly produce IL-4 and IL-10. Regulatory or suppressor T cells, represent potentially a major barrier to successful anti-tumor immune responses. These include Natural Killer T cells[7], CD25+CD4+ T cells [8,9] and Th3 cells[10]. The balance of signals processed by regulatory T cells can determine vastly different scenarios in tumor surveillance [11]. In the mouse system, CD25+CD4+ regulatory T cells suppress the activation and proliferation of other CD4+ and CD8+ T cells specific for auto antigens which of course is important to prevent autoimmunity but on the other hand prevents the effective generation of immunity to tumor antigens.

The rules that govern the balance between immunity and tolerance is controlled by the conditions of antigen encounter and activation status of the antigen presenting cell [10,12]. In general, systemic and persistent exposure of T cells to antigen in the absence of costimulation tends to result in T cell tolerization. The type and level of costimulation received during the first encounter with antigen are key determinants in the outcome of an immune response. This depends largely on the activation status of the professional antigen presenting cell that presents the antigenic peptide to naive T cells, in most cases the dendritic cell. The costimulatory state of professional antigen presenting cell is promoted by activated CD4+ T cells, in particular by interaction between CD40L on Th cells and CD40 on the APC [13-16]. This type of T cell help is essential for CTL induction under noninflammatory conditions, whereas lack of CD4+ T cell help can lead to CTL tolerization[17]. Direct demonstration that the activation status of antigen presenting cells influences the outcome of antigen recognition by CD8+ T cells was obtained in studies in which vaccination with mature dendritic cell induced cytotoxic T lymphocyte immunity, whereas infusion of immature dendritic cells failed to do so [15,18]. The conditions involved in setting the balance between tolerance and immunity seem to be different for activated T cells, because circumstances that tolerize naive T cells may not be tolerogenic for memory T cells. More details on the cellular immune system are to be found in recent reviews [19-22])

## Ovarian cancer and the immune system

While the interaction between the host immune system and ovarian cancer tumor cells is still not completely understood, several observations suggest that cell-mediated immune responses could be important in controlling ovarian cancer.

As already stated, the presence of antigen presenting cells, most favorable dendritic cells, is crucial in activating the immune system. In cancer patients the number of dendritic cells is decreased and functionally suppressed by the tumor microenvironment, inhibiting immune responses and thereby causing an impaired tumor immunity [23-27]. For several tumor types it was shown that the number of infiltrating dendritic cells correlated with good prognosis. In a retrospective study using immunohistochemistry the same phenomenon was observed in ovarian cancer [28]. The potential role of dendritic cells in ovarian cancer was demonstrated by Schlienger et al[29]. In 50% of ovarian cancer patients dendritic cells derived from peripheral blood mononuclear cells could, in vitro, induce tumor specific T cells upon loading the dendritic cells with tumor antigen derived from autologous tumor. The antigen(s) recognized by these T cells were not defined. Dendritic cells derived from peripheral blood mononuclear cells and tumor associated macrophages obtained from ascites from the same ovarian cancer patients, cultured with IL-4, GM-CSF and TNF-α, comparably stimulated T cell lines[30]. In contrast to the beneficial effects of macrophages and dendritic cells on the tumor specific immune responses, tumor associated macrophages have been shown to secrete the immunosuppressive cytokine IL-10[27,31]. One of the effects of IL-10 is that it induces B7-H1 expression on myeloid derived dendritic cells [32]. B7-H1, belonging to the B7 family of costimulatory molecules, is thought to be involved in the regulation of cellular immune responses through its receptors on activated T and B cells [33,34]. B7-H1 was first described to be expressed by ovarian cancer cells. Later it has been shown to be also present in other human carcinomas [33]. Tumor associated B7-H1 induces apoptosis of activated antigen specific T cells, contributing to the immune evasion of tumor cells [35]. Not only the ovarian cancer tumor cells but also myeloid derived dendritic cells obtained from ovarian tumor tissue and their draining lymph nodes express B7-H1, and are capable to downregulate T cell responses[32]. INF-γ upregulates B7-H1 on the surface of tumor cell lines [35], which might have implications for IFN-γ based cancer immunotherapy. To deal with this issue one could consider blockade of the B7-H1 pathway by e.g. neutralizing mAb. The efficacy of this approach has been shown very nicely in a mouse model for squamous cell carcinoma [36].

In ascites and tumors from patients with ovarian cancer myeloid dendritic cells are outnumbered by plasmacytoid dendritic cells [27,37,38]. The exact role of the plasmacytoid dendritic cells in priming naive T cells needs to be further elucidated. It seems that plasmacytoid dendritic cells produce high levels of the angiogenic cytokines TNFα and IL-8 in contrast to the myeloid dendritic cells which produce cytokine IL-12, an inhibitor of angiogenesis. Thus, the accumulation of plasmacytoid dendritic cells in ascites and ovarian cancer tumors is of benefit for the vascularization of the tumor and thereby promotes tumor growth[39].

In ovarian cancer tumor infiltrating CD4+ and CD8+ T cells have been studied extensively. MHC restricted tumor infiltrating lymphocytes cell lines and clones have been developed from lymphocytes derived from ascites and solid tumors of patients with ovarian cancer [40-44]. A clear association between tumor infiltrating lymphocytes and clinical outcome in ovarian cancer patients has been reported in a landmark paper by Zhang et al[45]. In a large cohort of 186 ovarian cancer patients, the five year survival rate was 38% among patients whose tumors contained T cells and only 4,5% among patients whose tumors contained no T cells. The presence of intratumoral T cells was an independent prognostic factor in a multivariate analysis. One of the other remarkable observations from this study was the correlation between high vascular endothelial growth factor expression and low number of T cells, suggesting that vascular endothelial growth factor reduces the number of T cells. T cells from patients with late-stage ovarian cancer contained increased proportions of regulatory CD25+CD4+ T cells, that secreted the immunosuppressive cytokine TGF-β[9]. In a very elegant study by Curiel et al it was shown that ovarian cancer tumor cells and associated macrophages produce the chemokine CCL22, which mediates trafficking of regulatory T cells in tumors and ascites but not to draining lymph nodes[46]. It was shown that these regulatory T cells suppressed tumor specific T cells and were associated with worse prognosis[46]. The regulatory T cells expressed high levels of CCR4, a receptor for CCL22. By blocking regulatory T cell attracting factors, like CCL22, patients might benefit to a higher extent of immunotherapeutic approaches. Also in the same paper by Curiel it was shown that HER-2/neu specific T cells were blocked by the regulatory T cells in their proliferative function, cytokine production and cytolytic activity. The papers of Zhang et al [45] and Curiel et al [46] seem to have conflicting results with Zhang et al showing a positive correlation between the presence of intratumoral T cells and survival and Curiel et al showing an inverse correlation. However in the first study the total number of T cells was taken into account and in the latter paper only the number of regulatory T cells. One can imagine that ovarian cancer patients with intratumoral T cells have a favorable prognosis as long as regulatory T cells are absent. Nevertheless, it will be important that the data from Zhang et al will be confirmed by others to elucidate the role of intratumoral T cells in ovarian cancer. It has been proposed by Conejo-Garcia et al that the ligand "Letal" (lymphocyte effector cell toxicity-activating ligand), expressed by ovarian cancer tumor cells has a role in survival and expansion of tumor infiltrating lymphocytes [47]. Higher levels of tumor derived "Letal" correlated with stronger lymphocyte infiltration. The same group recently published on a new mechanism of tumor vasculogenesis involving vascular endothelial growth factor in cooperation with antimicrobial inflammatory peptides called β-defensins mediated by a new population of CD11c positive leucocytes (DC precursors) named by these group "vascular leucocytes"' [48,49]. These observations provide a role for the immune system in tumor angiogenesis and need further research to assess what the implications for the clinic could be.

Cytokines and their role in the normal ovary and in ovarian cancer is nicely reviewed by Nash et al[50] and will not be discussed extensively in this review. Ovarian cancer cells probably only partially retain the ability to produce cytokines with important immunostimulatory functions, that are expressed by normal ovarian epithelial cells but lost during neoplastic transformation e.g. the pro-inflammatory cytokine IL-18 [51]. Stat3, a mediator in inflammatory responses and overexpressed in ovarian cancer [52,53], might play an important role in this change in cytokine production by tumor cells suppressing proinflammatory cytokine production[54].

MHC class I down regulation, an often observed immune escape mechanism in different types of cancer, has not been described frequently for ovarian cancer [55-57]. However recently, Vitale et al showed that MHC class I down regulation was associated with higher stage of disease, yet in a multivariate analysis not with survival [58].

The influence of cytoreductive surgery and platinum/paclitaxel based chemotherapy on the immune system in ovarian cancer has not been elucidated up to now. Whether the anti-tumor reactivity in ovarian cancer patients is influenced by surgery and / or chemotherapy remains to be determined. The immunogenicity of dying tumor cells upon chemotherapeutical treatment, does depend on the nature of the cell death (apoptosis or necrosis), but probably as important are local environment and the activation state of the dendritic cells. Platinum based chemotherapy induces apoptosis of ovarian cancer tumor cells. It is therefore encouraging that dendritic cells loaded with autologous apoptotic tumor cells are capable to induce strong tumor specific T cell responses[29]. T cells themselves are susceptible to chemotherapy [59], but high expression of "Letal" by tumor cells protects lymphocytes from cisplatinum induced cell death [47]. For tumor associated antigens like Mov18, OV-TL3 and OC125 only limited differences in expression on the cell surface of ovarian cancer cells were observed before and after chemotherapy[57].

## p53 as tumor antigen

### General introduction on p53

Specific T cell-mediated immunotherapy requires the identification of tumor-specific antigens carrying T cell epitopes presented in the context of MHC class I and/or MHC class II molecules (reviewed by[19,20,60,61]) An attractive tumor specific antigen in ovarian cancer is the frequently overexpressed and mutated p53 protein. Other possible target antigens like HER-2/neu and MUC-1 are less frequently expressed by ovarian tumor cells. P53 is a tumor suppressor protein. The role of p53 and other cancer genes has been reviewed by Vogelstein and Vousden [62-64]. P53 acts as a transcription factor, playing a key role in coordinating cell cycle arrest, DNA repair and apoptosis following DNA damage to promote genomic stability. P53, as a transcription factor, mediates apoptosis by pathways involving the upregulation of pro-apoptotic genes as well as downregulation of anti-apoptotic genes [65]. P53 also has the capacity to induce apoptosis directly from the cytoplasm via direct activation of Bax to permeabilize mitochondria which will release cytochrome c leading to the induction of apoptosis [66]. In cancer cells loss of wild-type p53 function may lead to more aggressive tumor growth and failure to respond to standard therapy. The most common way of loss of function is through mutation. P53 is one of the most commonly mutated tumor suppressor proteins in human tumors [67], and already more than 4000 different mutations have been described. The majority are point mutations, resulting in single amino-acid substitutions, generally occurring in the central region of the protein (amino acid 100–300). Other tumor suppressor genes often lose their expression after mutation, but the point mutated p53 protein is often more stable and therefore overexpressed in tumor cells. The loss of function of p53 might be due to binding of the mutated protein to the wild type protein (non-functional tetramers) or to loss of the wild type allele (loss of heterozygosity) [67,68]. P53 mutations are associated with poor prognosis. Other ways of inactivation include binding to overexpressed MDM2 or E6 protein of human papillomavirus, both causing rapid p53 protein degradation via the ubiquitin pathway[62,63]. Increased resistance to chemotherapy by mutant p53 has been linked to loss of the presumed triggering role of wild-type p53 in the process of apoptosis.

## P53 as tumor antigen (preclinical studies)

P53 protein is overexpressed in 50–60% of ovarian cancers [69-73]. Restoration of the function of p53 in tumor cells is one therapeutic approach. Important progress has been made recently in this field, using viral and non-viral vectors [74], or p53 activating peptides [75]. On the other hand, p53 seems an attractive target for cancer immunotherapy. Due to mutation, nuclear and cytoplasmatic levels of p53 are strongly increased in tumor cells compared to normal cells, thereby providing an immunological window for p53 wild-type specific immune effector cells [76,77]. Still, tolerance against an autoantigen as wild type p53 needs to be overcome, without development of autoreactive T cells. Mutant and wild-type p53 specific CTL have been described in mice [78-85] In mice, eradication of tumors was achieved with vaccines composed of p53 wild type and mutant peptides [81-83], as well as with adoptive transfer of wild type p53 specific T cells [78,85-87]. To immunize with whole p53 protein expressed by e.g. viral vectors or long peptides overlapping a whole protein has the advantage of multiple MHC class I and II restricted epitope expression (dominant as well as cryptic). Mouse dendritic cells transduced with an adenoviral wild type p53 encoding construct generated wild type p53 specific CTL (after i.v. or s.c. immunization) capable of preventing the outgrowth of sarcoma tumors[88,89]. Moreover, the same construct used intratumorally, induced a systemic antitumor response against p53 overexpressing tumors, despite the fact that anti p53 T cell responses could not be measured[90]. Intratumoral injections with recombinant canarypox virus expressing wild type murine p53 (ALVAC-p53) showed antitumor effects in 66% of the mice, however without detectable anti p53 CTL responses [91]. Using different routes of ALVAC-p53 immunizations only intravenous administration was capable of inducing anti-p53 CTL response [92]. More successful than the ALVAC-p53 immunizations in mice was the approach using a recombinant modified vaccinia virus Ankara, expressing wild-type murine p53 (MVAp53). This cell free immunization strategy protected mice for the outgrowth of a syngeneic murine sarcoma by intraperitoneal injection of MVAp53[93]. Mice immunized s.c. with a recombinant vaccinia virus construct expressing wild type p53 were protected against challenge with a p53 overexpressing glioblastome cell line (GL261). Achieving successful p53 based immunization in the presence of well established tumors probably requires active adjuvants. CTLA-4 plays an important role in (negative) regulation of T cell responses [94]. The p53 specific CTL and Th responses can be enhanced by using anti-CTLA-4 at the time of antigenic stimulation, thereby even more effectively breaking tolerance [93,95]. Anti-CTLA-4 blockade in combination with a vaccine adjuvant, CpG ODN (synthetic oligodeoxynucleotide containing unmethylated cytosine-phosphate-guanine motifs) had a synergistic effect on the improvement of MVAp53 induced antitumor immunity[96]. Using MVAp53 based immunization Dafterian et al showed eradication of large, well established tumors in three different tumor models in two different strains of mice[96]. The immune response against p53 can also be enhanced by the activation of CD40 [89,97]. Triggering of the CD40 receptor on dendritic cells is vital for their adequate activation and maturation. Both compounds, anti-CTLA4 and activators of CD40, will become available to test on a wide-based scale in clinical studies within the near future. Another route of enhancement of p53 specific immune response after immunization was obtained by administration of Flt3 Ligand, a strong DC stimulating adjuvant[98]. High steady state levels of p53 are not a pre-requisite for tumor eradication by p53 specific CTL as mentioned in one study[99]. Instead, p53 turnover is an important factor in determining the sensitivity of tumor cells to these CTL [87,100]. CD4+ T helper cells are crucial in the recruitment and regulation of the innate and adaptive immune effector cells[101]. We have demonstrated that CD4+ p53 specific T-helper cells are able to help tumor-specific CTL in controlling p53 overexpressing tumors [102]. Using MHC-transgenic mice has shown to be very efficient in obtaining MHC class I restricted CTL against p53 with high avidity capable of lysing p53 overexpressing tumor cells without lysis of normal cells expressing normal levels of p53 [77]. Very elegantly Kuball et al showed that a CD8-independent p53 specific T cell receptor, generated in HLA A2.1 transgenic mice, could be expressed in human CD8+ and CD4+ T cells with p53 specific tumor recognition[103]. This is at least a very efficient way to obtain p53 specific class I restricted T cells with very high affinity. These model systems might help to answer questions on self tolerance for tumor antigens like p53 and intriguing aspects like cross presentation, cross priming and different aspects of immunotherapy in cancer. So far neither clinical nor immunopathological damage to normal tissue has been observed in different mouse models, despite the fact that wild type p53 is expressed in normal tissue. This indicates that p53 specific T cells are truly tumor-specific. Data available so far support the view that p53 specific immunotherapy may offer a wide therapeutic margin in cancer patients. Proof of the pudding is still in the eating, knowing that their might be important differences in the immune system between preclinical models and men as nicely reviewed by Mestas et al [104].

Cicinnati et al studied the potential of prophylactic vaccination with p53 epitopes using DNA and /or peptide pulsed dendritic cell vaccination in the tumor model giving rise to sarcomas[105]. Compared to control mice a higher incidence of epitope loss tumors were detected in the prophylactic vaccinated group resulting in an increase in tumor growth. Vaccine induced tumor escape therefore could be an important risk in p53 based prophylactic vaccines.

## P53 as tumor antigen (clinical studies)

In humans MHC class I restricted p53 specific CTL [106-121], MHC class II restricted p53 specific proliferating Th cells [122-125], and p53 antibody responses (summarized in Table 1) have been observed [123,126-133]. The first phase I/II immunization trials using p53 as an antigen have just finished and new trials are being initiated. In a phase I study, six advanced stage cancer patients were immunized with an adenoviral vector encoding wild type p53[134]. Neither tumor responses nor anti p53 responses were observed, however all patients showed an adenoviral immune response. This strong anti adenoviral specific response may limit a p53 specific response. Based on the results in the mouse system[91,92,135] and rhesus macaques [136], a phase I/II clinical study involving vaccination of end-stage colorectal cancer patients with a recombinant canarypox virus (ALVAC) encoding wild type p53 was performed[137]. Patients were immunized intravenously with an increasing dosage of ALVAC-p53. From this study it appeared that this modality is safe and capable of stimulating p53-specific Th1 (IFNγ) responses in several of these patients. One out of 16 patients showed stable disease for a short period of time after immunization with the highest dose. Fever was the only vaccine related adverse effect. The authors conclude from this trial that repeated immunizations are probably necessary to obtain good clinical responses. Again, anti-vector responses were observed in all patients after vaccination which might have impaired the anti-p53 immune responses. Preclinical data have shown the superiority of prime and boost vaccine strategies using different viral vectors [138,139]. Whether or not the route of administration plays a role is under debate[140]. Clinical studies have shown the safety and effectiveness of prime and boost vaccination protocols using different viral vectors to deliver the antigen of interest[141,142]. An analysis of the p53 specific Th response before and after surgery for colorectal cancer showed that the majority of the Th responses detected were not associated with the immunostimulatory cytokine IFNγ, whereas a number of Th responses even involved secretion of the immunomodulatory cytokine IL-10, pointing at the activity of T-regulatory cells that are known to suppress T cell immunity[143]. These results more or less resemble the cytokine profiles of tumor associated T cells derived from ovarian tumors, which were also associated with a lower zeta chain expression[144]. It is important to further investigate the character of the p53 specific T cell responses, because p53-based vaccination of patients should be aimed at boosting only the desired Th1-type immunity, while stimulation of T-regulatory cells should be avoided. This finding would argue in favor of application of a p53-specific vaccination using a delivery mode specifically stimulating the anti p53 (cytotoxic T cell and) Th1 responses. Autologous dendritic cells expressing the antigen of interest is one of these ways. Svane et al reported on their phase I immunization study in breast cancer patients with p53 peptide pulsed DC[145]. Dendritic cells were pulsed with three wild-type and three modified HLA-A2 restricted p53 peptides combined with a MHC class II binding peptide (PADRE). Patients received ten subcutaneous immunizations with at least 5 × 10^6 ^peptide pulsed dendritic cells combined with 6 mIU/m^2 ^IL2. Two out of six patients had a clinical response and three out of six had p53 specific T cell responses (including the two patients with a clinical response), without inducing significant toxicity. Another vaccination strategy would be the use of long peptides encoding the whole protein of interest. The advantage of using long peptides is that, if delivered in the appropriate adjuvant (with dendritic cell stimulatory capacity), all potential MHC class I and class II epitopes within the delivered peptides will be processed and presented to host T cells. Table 2 and 3 summarize the naturally processed wild-type p53 epitopes in MHC class I and II known so far. These vaccines will thus become independent of MHC binding motif prediction or processing algorithms and can be administered to subjects independent of their MHC type. A phase I – II trial using wild- type p53 derived long peptides in ovarian cancer patients will be initiated at the University Medical Center Groningen in 2005.

**Table 1:** Serum p53 antibodies in patients with epithelial ovarian cancer.

Reference	Total no of patients	No of patients with p53 serum antibodies (%)			Correlation with overall survival
		In all patients	In patients with stage I/II disease	In patients with stage III/IV disease	
			
[146]	86	18 (21)	3 (10)	15 (27)	no^1^
[131]	113	21 (19)	3 (8)	18 (23)	yes^1,2^
[147]	83	38 (46)	5 (26)	33 (52)	no^2^
[148]	193	24 (12)	4 (6)	20 (15)	no^1,2^
[149]	33	12 (36)	3 (21)	9 (47)	yes^1^
[150]	30	10 (33)	2 (22)	8 (38)	-
[151]	174	41 (24)	8 (21)	29 (28)	no^1,2^
[133]	113	28 (25)	-	-	no^1^
[152]	99	25 (25)	-	-	-
[127]	46	4 (9)	-	-	-
[130]	30	8 (27)	-	-	yes^1^
[153]	30	8 (27)	-	-	-
[129]	40	15 (38)	-	-	-
[126]	46	4 (9)	-	-	-
[154]	38	11(29)	-	-	-

	1154	267 (23)	28 (13)	132 (28)	

1: tested in an univariate analyses. 2: tested in a multivariate analyses.

**Table 2:** Naturally processed human wilt-type p53 derived epitopes in MHC class I

Allel	amino acid nr.	Sequence	Reference
HLA-A*0201	65–73	RMPEAAPPV	[115,155]
HLA-B*4601	99–107	SQKTYQGSY	[117]
HLA-A2	103–111	YQGSYGFRL	[120]
HLA-A24	125–134	TYSPALNKMF	[156]
HLA-A2	139–147	KTCPVQLWV	[120,157]
HLA-A2.1	149–157	STPPPGTRV	[84,124]
HLA-A*0201	187–197	GLAPPQHLIRV	[115]
HLA-A2	217–225	VPYEPPEVG	[118]
HLA-A*0201	264–272	LLFRNSFEV	[84,111]

**Table 3:** Naturally processed human wilt-type p53 derived epitopes in MHC class II

Allel	amino acid nr.	Sequence	Reference
HLA-DR1/HLA-DR4	108–122	GFRLGFLHSGTAKSV	[158]
HLA-DRB1*0401	110–124	RLGFLHSGTAKSVTC	[124]
HLA-DP5	153–165	PGTRVRAMAIYKQ	[125]
HLA-DRB1*1401	193–204	HLIRVEGNLRVE	[125]

## Conclusion

Progress in the fight against ovarian cancer has been hampered by the lack of highly effective therapy to permanently eradicate disseminated intraperitoneal metastases, which are present in most patients at the time of diagnosis. In order to improve the poor outcome for ovarian cancer patients standard and new treatment modalities, such as targeted or biologic agents and immunotherapy should be combined. In this review we pointed out that ovarian cancer tumor cells may (over)express immunoregulatory molecules such as ligand "Letal", CD40 and Stat-3 which stimulate immune response. On the other hand molecules are expressed which downregulate MHC class I molecules and / or simultaneously produce ligands such as CCL22 attracking regulatory T cells as immune-escape mechanism. Recent data showing the importance of the immune response in the course of ovarian cancer and the availability of new potent immunization strategies urge further exploration of immunotherapy as adjuvant treatment modality in ovarian cancer patients. The immune response against p53 can be enhanced by the activation of CD40, anti CTLA-4 blockade, coadministration of Flt3 Ligand and CpG ODN. Compounds capable of activating or blocking these molecules will become available within the near future to be tested on a wide-based scale in clinical studies. The role of p53 as tumor antigen in ovarian cancer in immunotherapy based trials will be unravled within the near future as well. Next to important issues as safety and immunogenicity of vaccination strategies, clinical effectiveness should be one of the major aims of future trials.

HW Nijman is supported by the Dutch Cancer Society (Grant nr. 2002-2768)
